# Supplementary figures and images for: SULF1 suppresses Wnt3A-driven growth of bone metastatic prostate cancer in perlecan-modified 3D cancer-stroma-macrophage triculture models
Source: PLoS One. 2020 May 15;15(5):e0230354. doi: 10.1371/journal.pone.0230354 (PMC7228113; doi:10.1371/journal.pone.0230354)

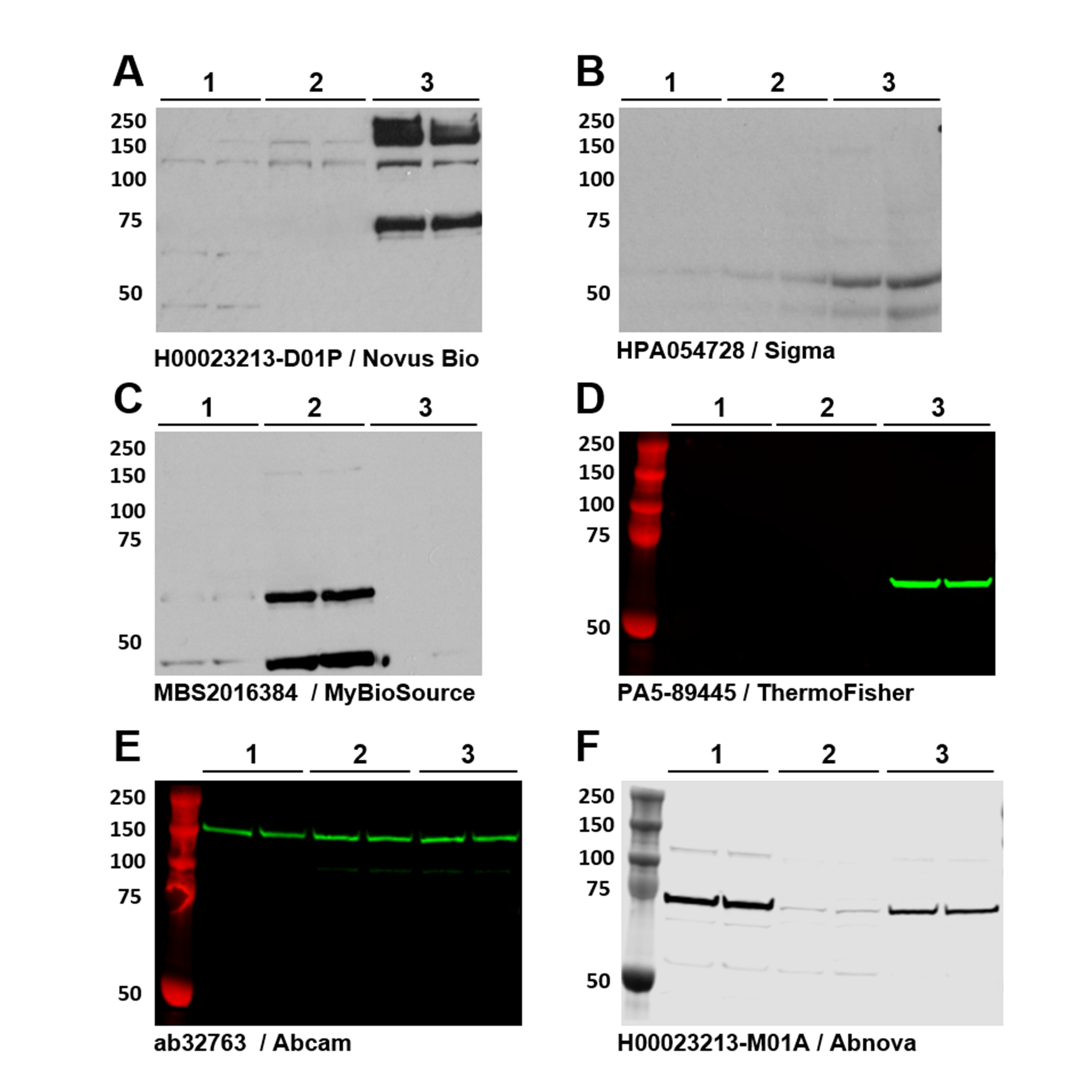

Supplement: S2 Fig — In blots from A-F: 1—SULF1-KO-HS27A cells, 2—WT-HS27A cells, and 3—C4-2B cells. Western blot was conducted as described in Materials and Methods. Approximately 20 μg of total protein lysate was loaded per well. All the antibodies tested are listed under each blot. All antibodies show one or multiple bands, all of which are inconsistent with the predicted molecular weight of 100-125 kDa for SULF1. The amounts used were equal to the highest concentrated dilution recommended by the manufacturers. Blots A-C were developed via enhanced chemiluminescence and films were exposed for approximately one minute. Blots D-F were developed by fluorescence detection using secondary antibodies labeled with near infra-red flourophores, as described in Materials and Methods. The raw, uncropped images can be found in file “S1 Raw images”. (TIF) [file pone.0230354.s002.tif]

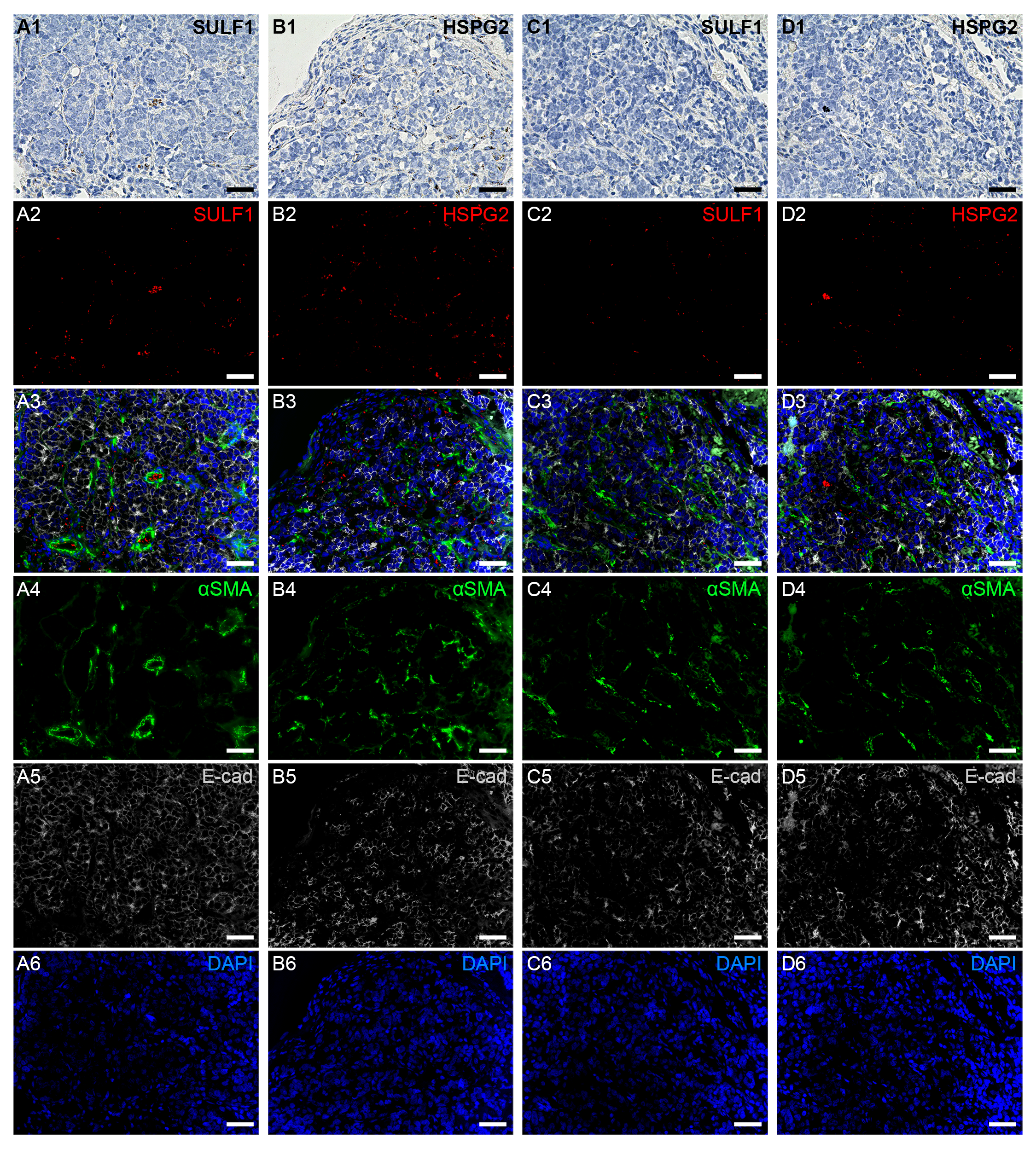

Supplement: S3 Fig — (A-D) RISH–immunofluorescence multiplexing of SULF1 (A1-6 and C1-6) and HSPG2 (B1-6 and D1-6) with the stromal marker αSMA (green) and epithelial marker E-cad (gray). A and B were magnified for display in Fig 2 and represent distinct regions of serial sections probed with the respective markers. C and D represent the same region of serial sections probed with the respective markers. The chromogenic signal in panels A1, B1, C1, and D1 was deconvoluted in ImageJ to create images A2, B2, C2, and D2, as described in Materials and Methods. RISH–immunofluorescence multiplexing was performed as described in Materials and Methods. Nuclei are stained with DAPI (blue). Scale bars correspond to 40 μm. Images were acquired using 40x objectives. Four additional images were acquired per sample, at random regions, which were used for quantification of SULF1 and HSPG2 signals, as described in Materials and Methods. (TIF) [file pone.0230354.s003.tif]

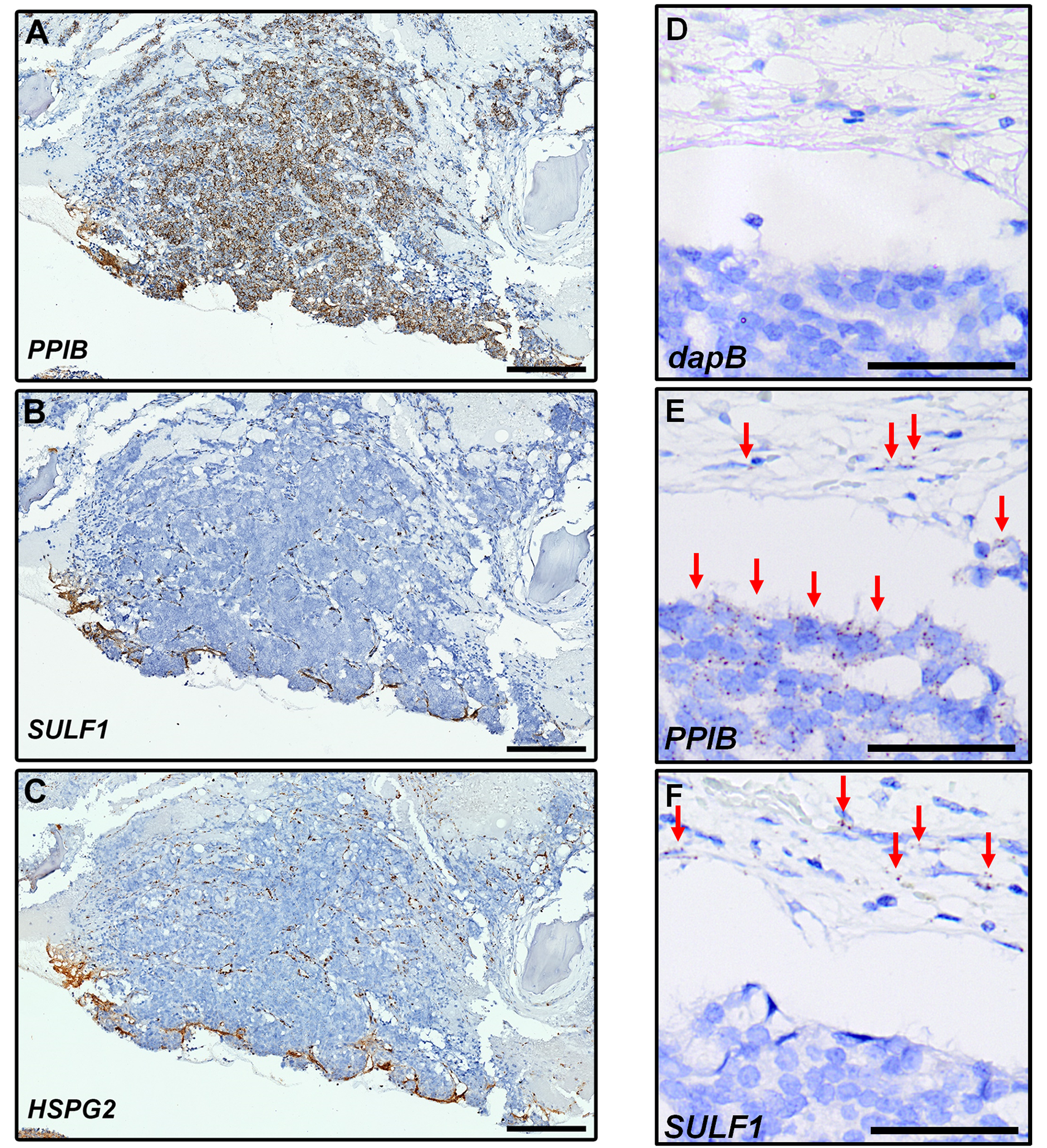

Supplement: S4 Fig — This figure shows the mRNA expression of the positive control gene, (A) Peptidyl-prolyl cis-trans isomerase B (PPIB), (B) SULF1, and (C) HSPG2 in the same region of cervical spine specimens. In D, E, and F, femur samples also are probed and include the negative control gene dihydrodipicolinate reductase (dapB). PPIB expression was widespread in all cells of the tissue, indicating good quality of the mRNA in the sample, whereas SULF1 and HSGP2 were generally confined to the stroma surrounding tumor nests. The PPIB control was used on every independent replicate experiment. The RNAscope assay was performed as described in Materials and Methods. Scale bar represents 200 μm for A-C and 50 μm for D-F. (TIF) [file pone.0230354.s004.tif]

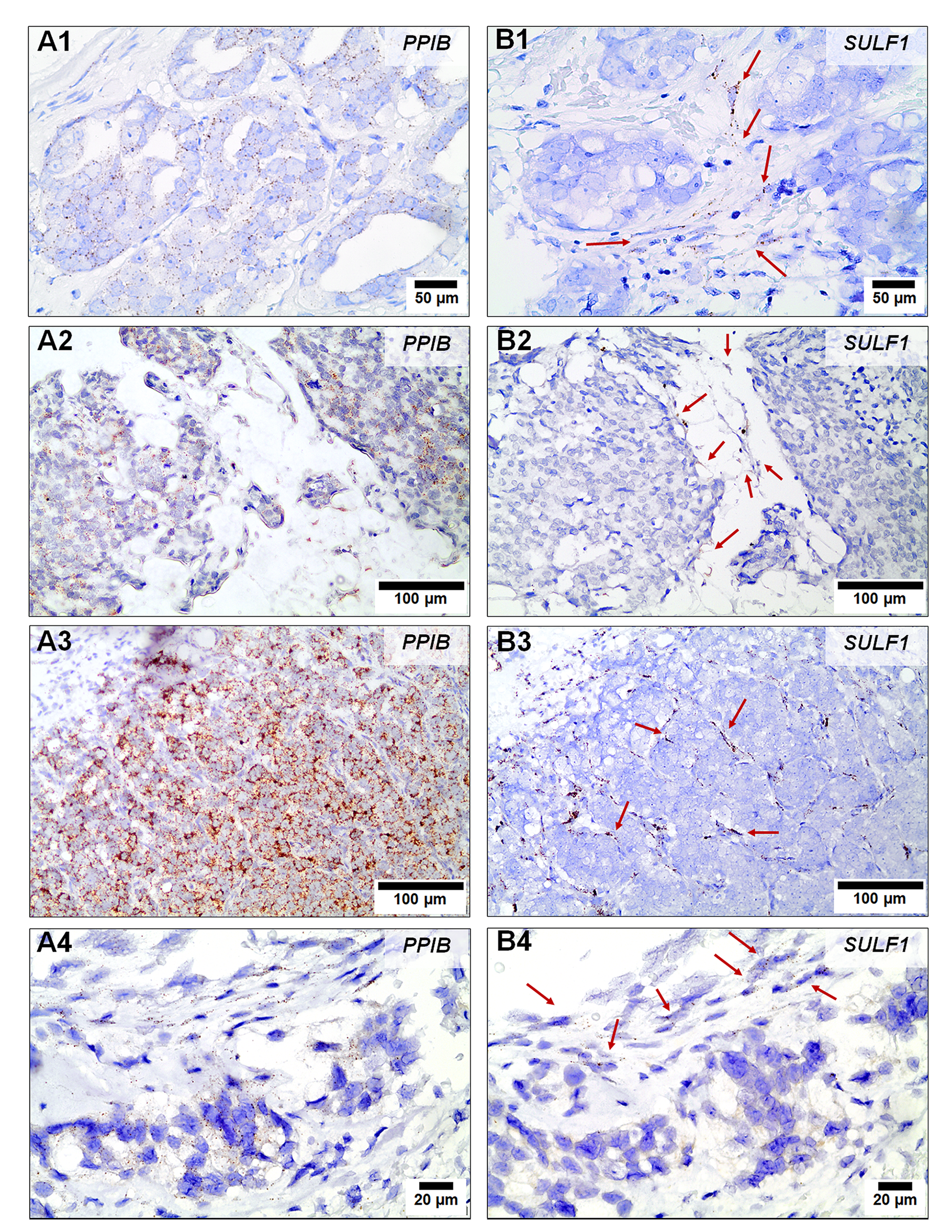

Supplement: S5 Fig — In situ hybridization for PPIB mRNA (A1-4) was performed in all hybridization experiments as a positive control for the assay. Samples which failed to show PPIB mRNA signal were disregarded for further analyses. As demonstrated above, SULF1 signal (B1-4) is mostly confined to the reactive bone marrow stromal cells, while PPIB is expressed throughout the serial sections. The tissues of origin for the samples used were femur (A1 and B1), cervical spine (A2, B2, A3, and B3) and acetabulum (A4 and B4). (TIF) [file pone.0230354.s005.tif]

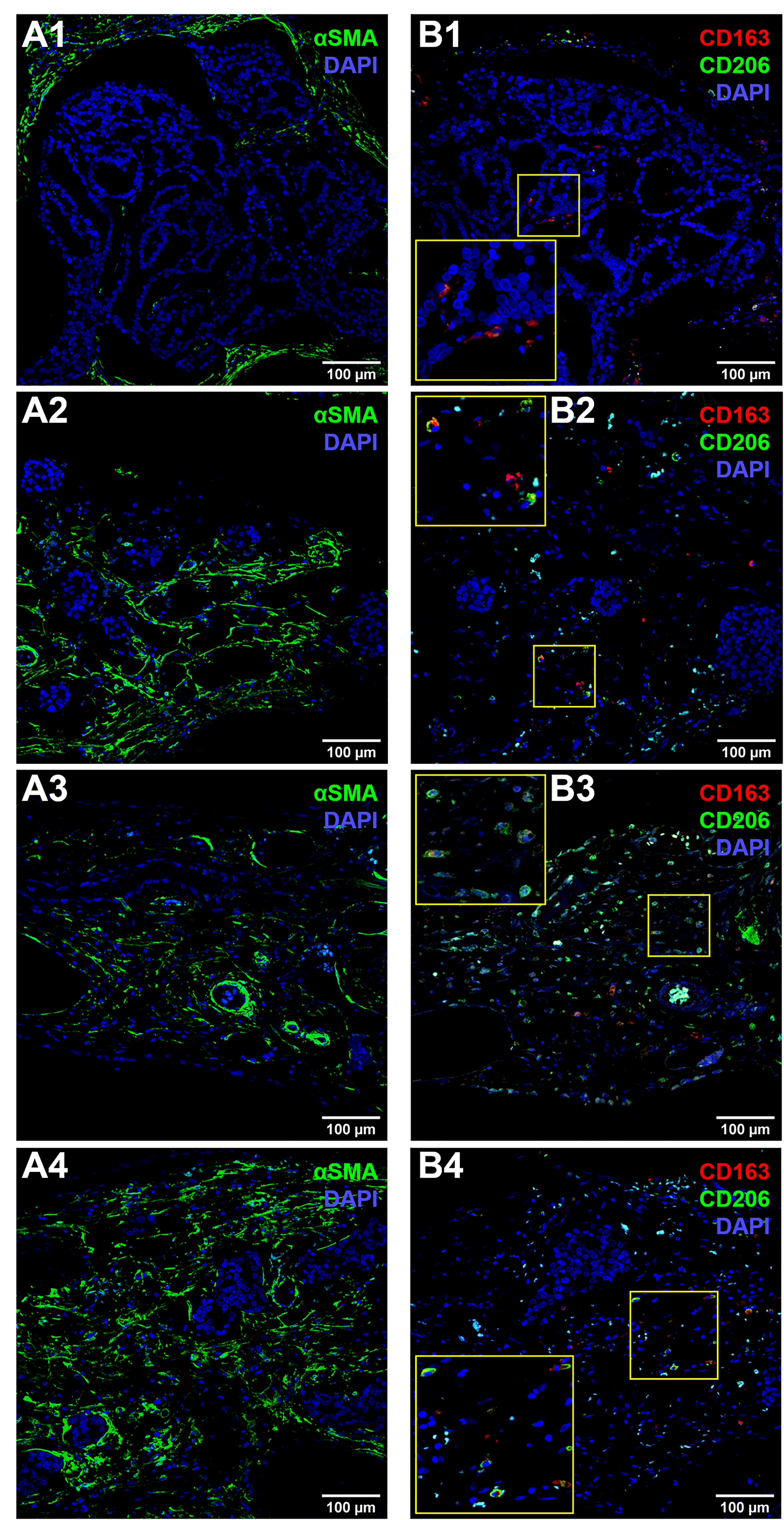

Supplement: S6 Fig — Immunofluorescence staining is shown for PCa bone metastasis samples from additional patients. As shown above, reactive bone marrow fibroblasts show strong αSMA signal (A1-4). The macrophage infiltration, indicated by the CD163 and/or CD206 staining (B1-4), varied depending on the patient, but the phenotype was generally consistent with polarization towards tumor-promotion (M2-like) macrophages. Larger yellow boxes indicate insets amplified from the smaller yellow boxes within the same figure. The tissues of origin for the samples used were femur (A1 and B1), cervical spine (A2, B2, A3, and B3) and acetabulum (A4 and B4). (TIF) [file pone.0230354.s006.tif]

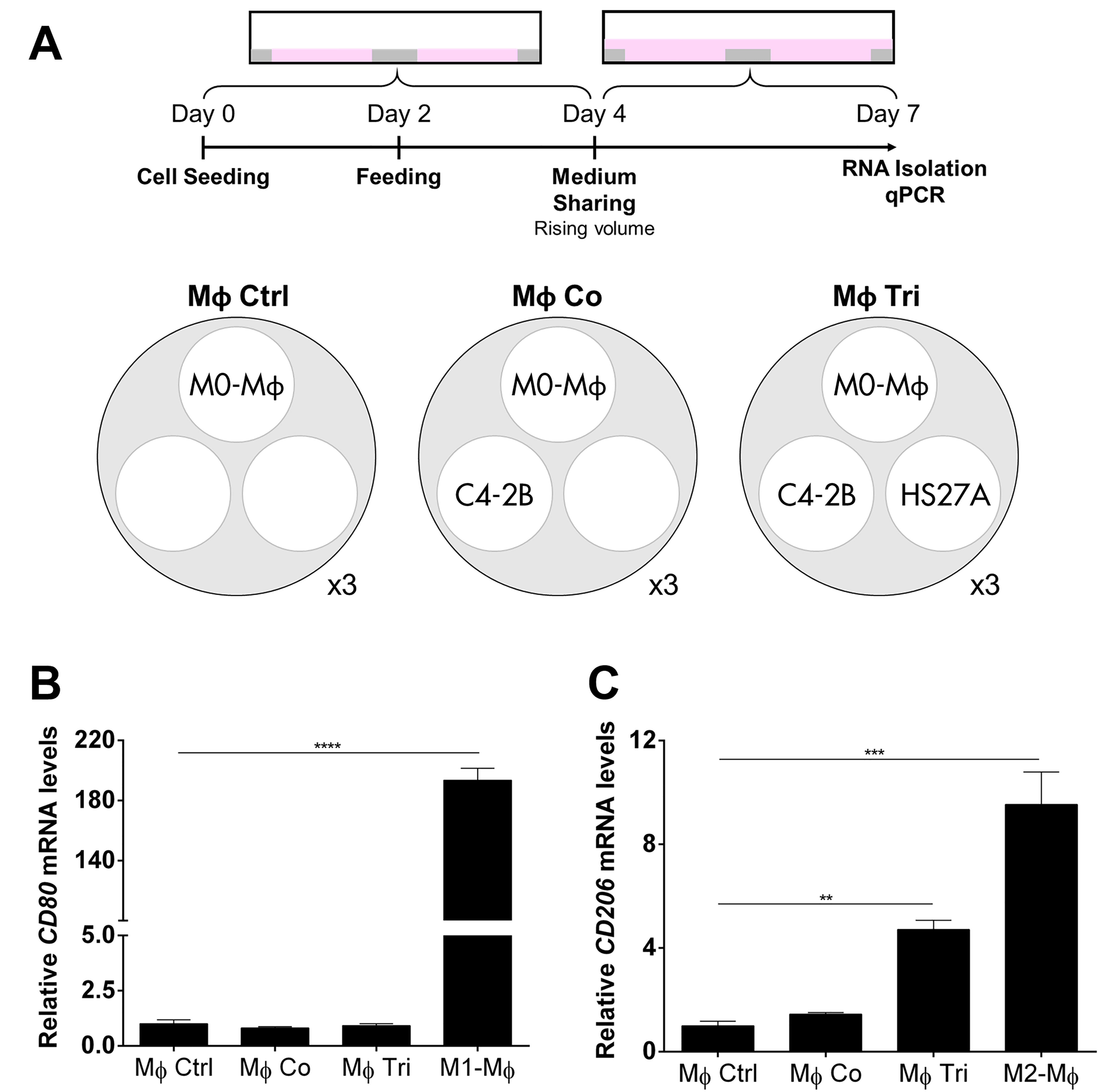

Supplement: S7 Fig — A. An indirect coculture system was designed in which a PDMS (grey) mold with laser-cut wells were placed in 100-mm dishes. The area of each well was 9 mm2 and the thickness of the mold was 3 mm. Culture combinations were as illustrated. As described in Materials and Methods, RNA was collected from unpolarized macrophages (Mϕ-Ctrl), and M1- or M2-polarized macrophages (M1-Mϕ and M2-Mϕ, respectively). B. Signals produced by C4-2B alone or C4-2B and HS27A cells did not drive macrophages towards the classically activated phenotype, indicated by CD80 mRNA expression. C. In contrast, while C4-2B cells alone could not induce up-regulation of CD206, factors produced by C4-2B and HS-27A cells up-regulated expression of CD206, indicating polarization towards a TAM-like phenotype. RNA isolation and macrophage polarization were performed as described in the materials and methods section. The expression of CD80 (B) and CD206 (C) was normalized to that of GAPDH. Values obtained for the Mϕ Ctrl group were arbitrarily set to 1 for comparison. Data shown represent the mean ±SD of two independent experiments. **, P < 0.01. (TIF) [file pone.0230354.s007.tif]

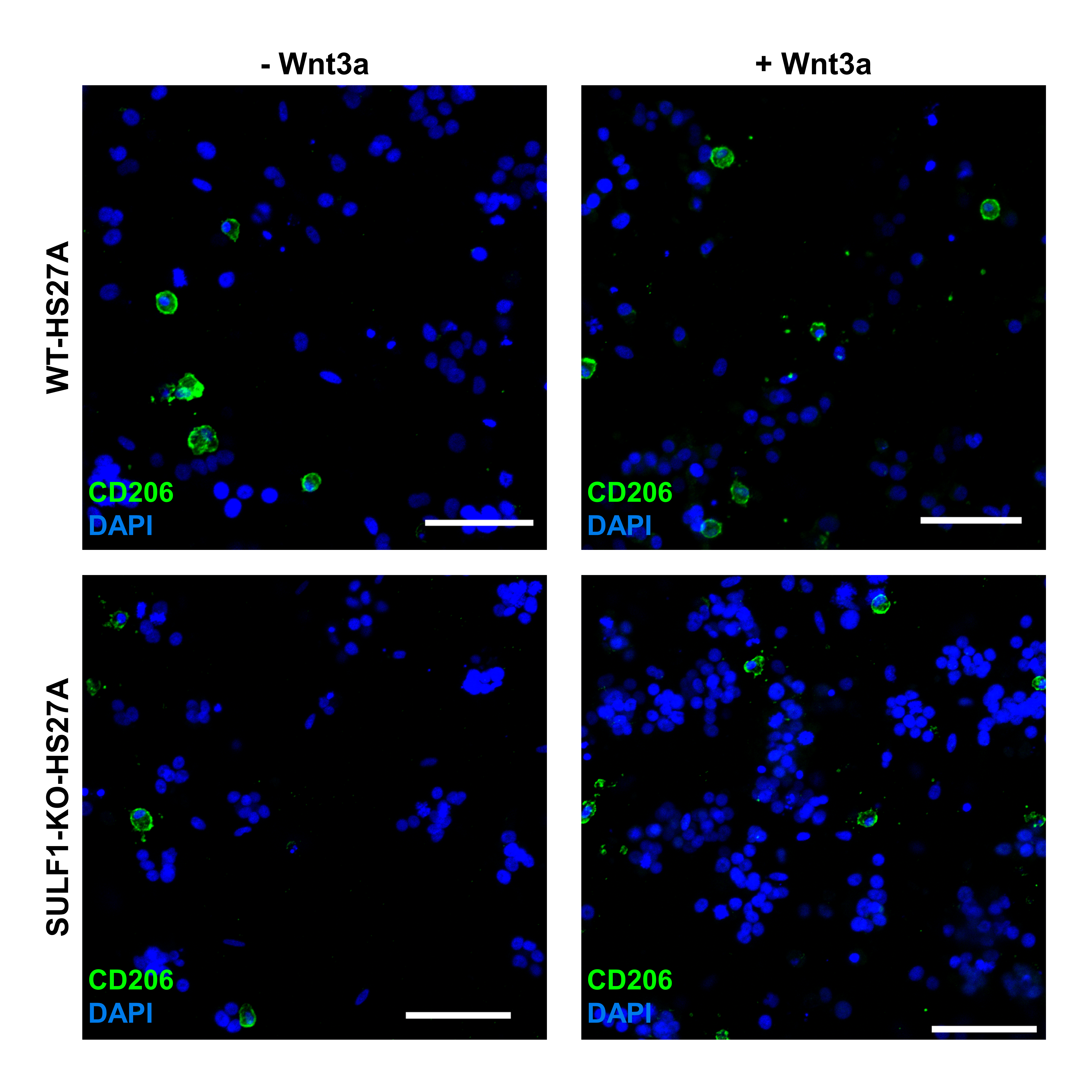

Supplement: S10 Fig — Biomimetic collagen-HA hydrogels were prepared as detailed in Materials and Methods, identically to the experiment in Fig 5. Immunofluorescence stainings show CD206 (green) with DAPI (blue) counterstain. With the CD206 signal, we created the parameters for the sphericity filter used in Fig 5A2, 5A4, 5A6 and 5A8, to pseudo-color macrophages in yellow. Scale-bar = 100 microns. Data shown represent the mean ±SD of three independent experiments. In each experiment, n = 5 hydrogels for each of the four groups. (TIF) [file pone.0230354.s010.tif]

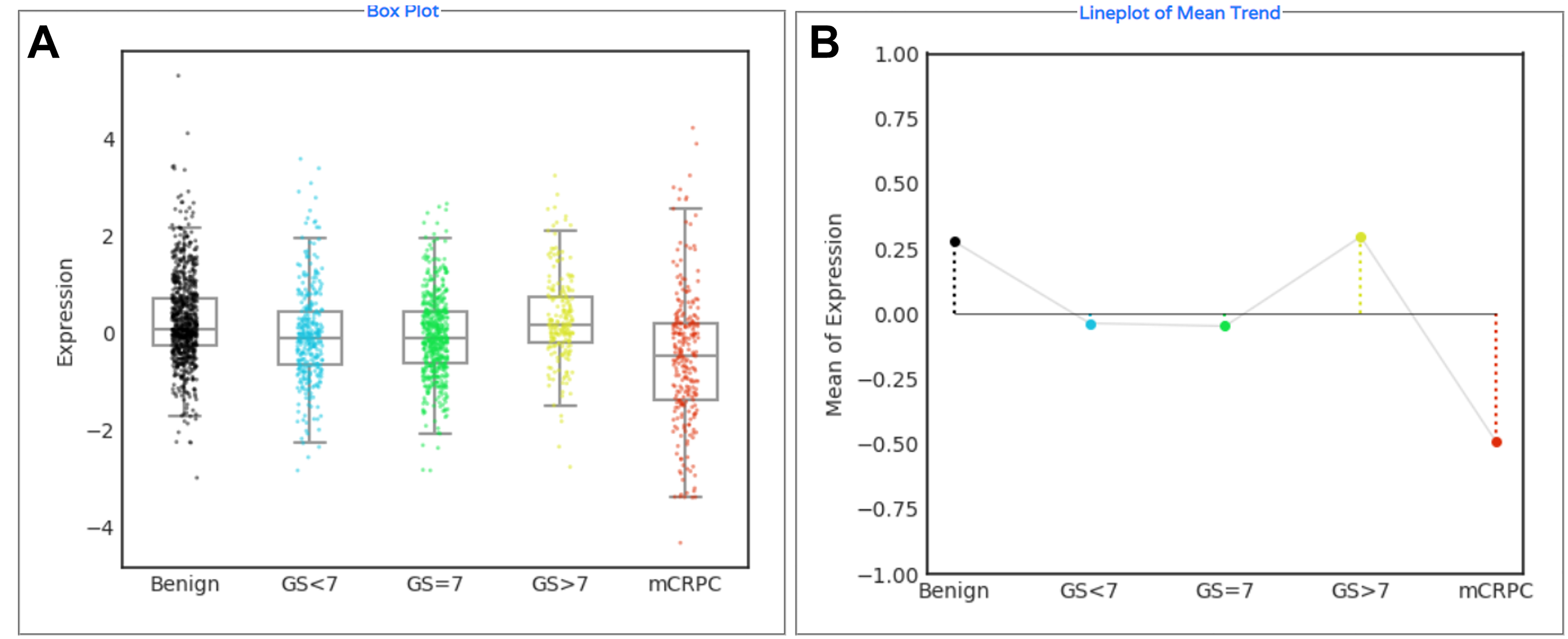

Supplement: S11 Fig — Expression data can be visualized via box plot (A) or lineplot of mean trend (B), which categorize the patient sample data from benign, local disease to increasing values for the Gleason Score (GS) and mCRPC. These data are consistent with reduction of SULF1 expression in the most advanced disease stage. (TIF) [file pone.0230354.s011.tif]

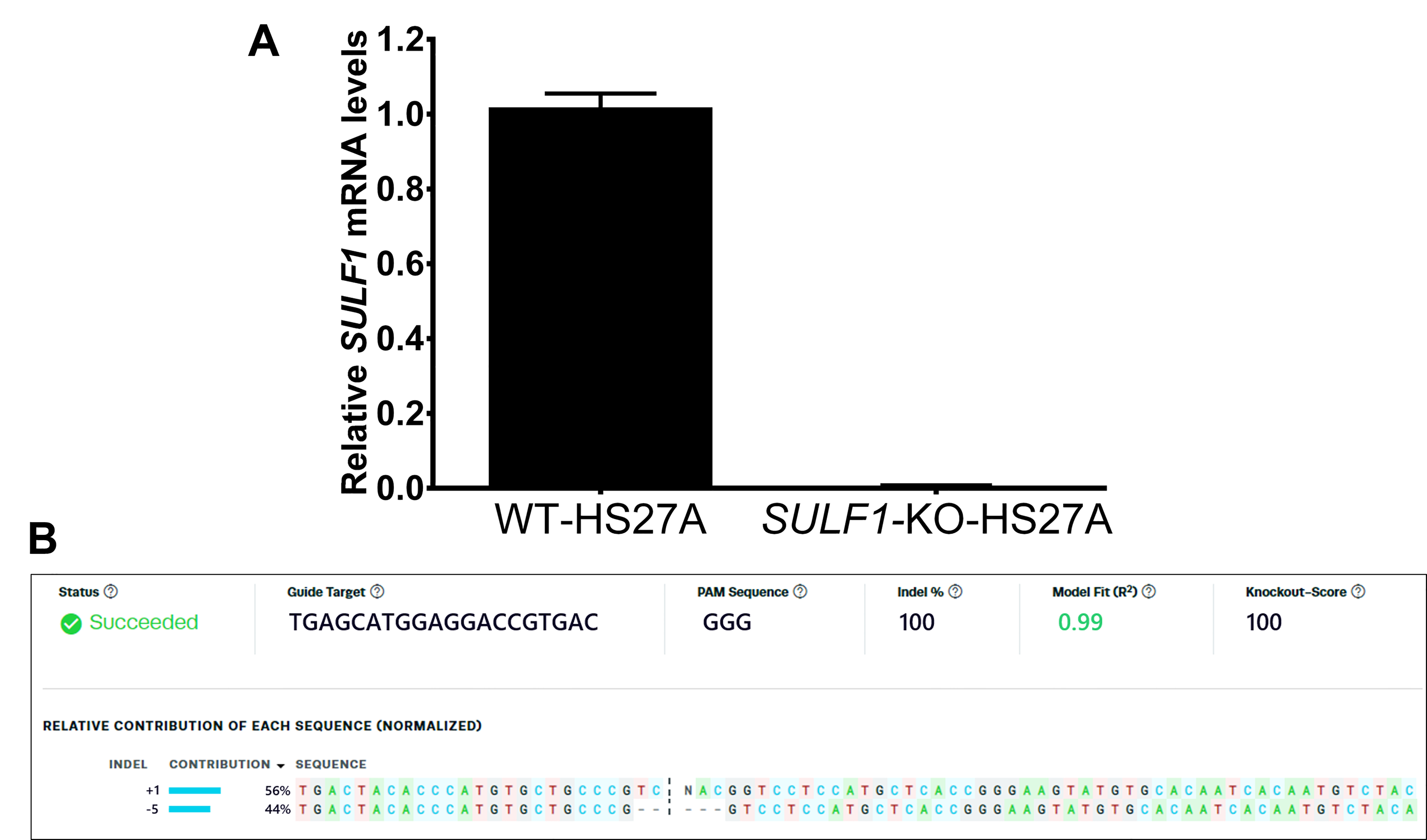

Supplement: S12 Fig — A. After isolation of multiple clones, we used qPCR to screen for SULF1 mRNA expression, as described in Materials and Methods. The most complete SULF1-KO-HS27A monoclonal population is described here compared to wild-type (WT) HS27A cells. B. DNA was extracted, from both WT and SULF1-KO-HS27A cells and sequenced around the CRISPR cut sites (represented by black vertical dotted lines). Sequencing primers were provided in the Gene Knockout Kit by Synthego. The contributions (%) show the inferred sequences present in the SULF1-KO-HS27A population. Indel % represents the percentage of sequences with mutations. Our results reveal only two sequences, showing +1 base pair (bp) and -5 bp indels. The Knockout-Score indicates the proportion of cells that have either an indel that causes a frameshift or 21+ bp indel. The score of 100 indicates a complete functional KO of SULF1 from HS27A cells. (TIF) [file pone.0230354.s012.tif]

**A**

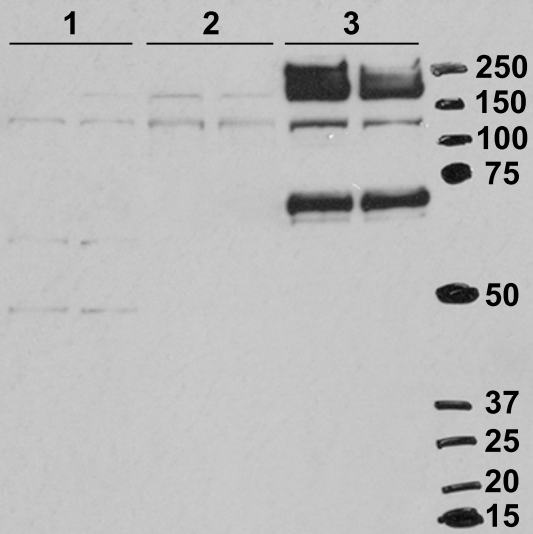

**B**

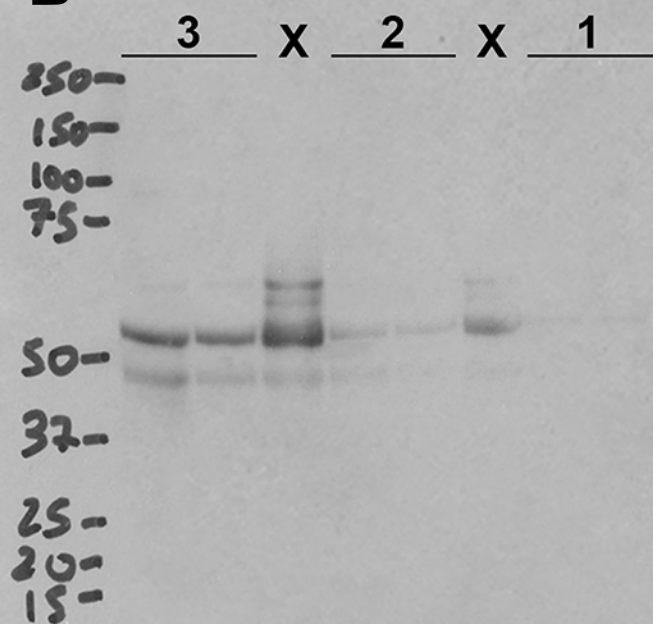

**C**

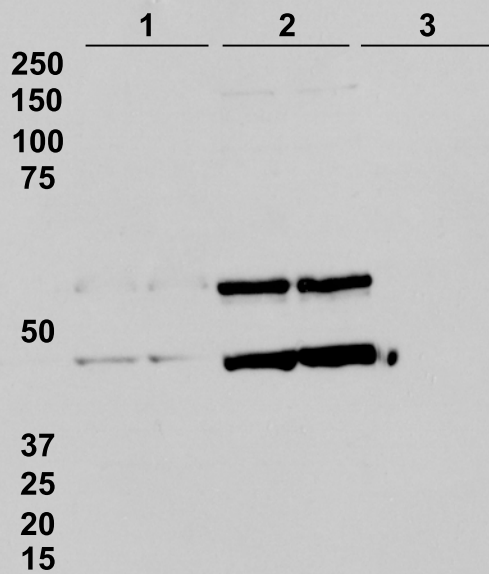

**D**123

X

X

250  
150  
100  
75  
50  
37  
25  
20  
15

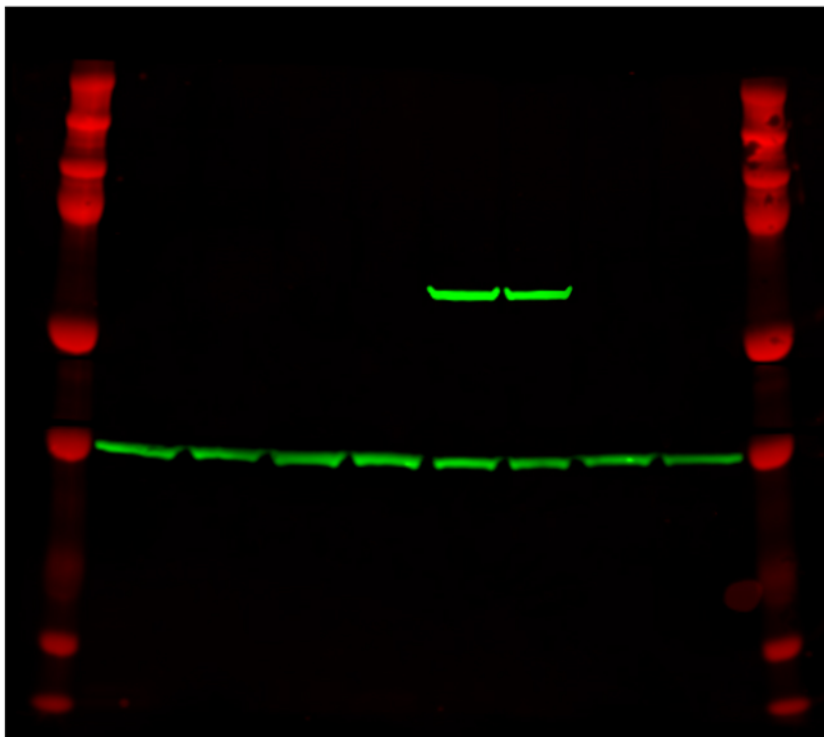

**E**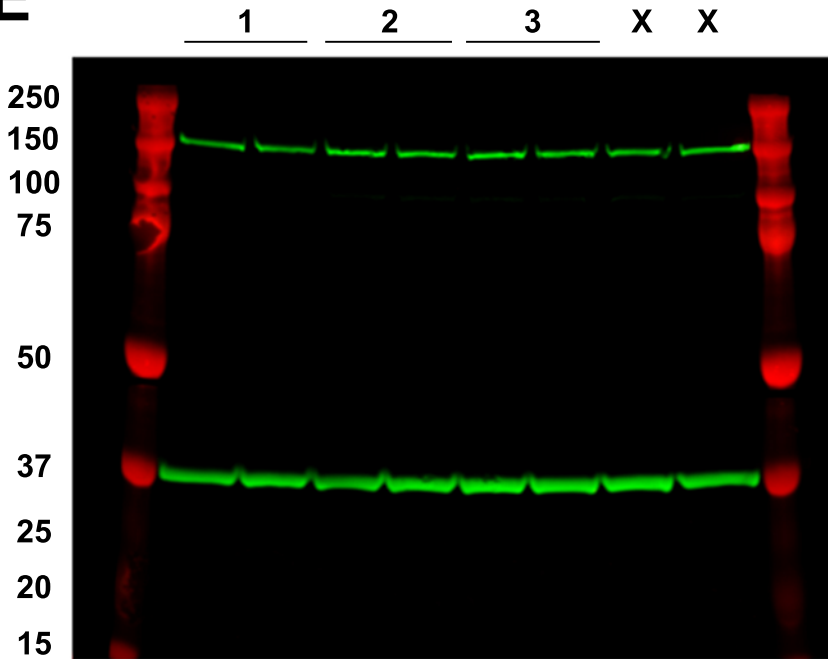

**F****X X****1****2****3****250****150****100****75****50****37****25****20****15**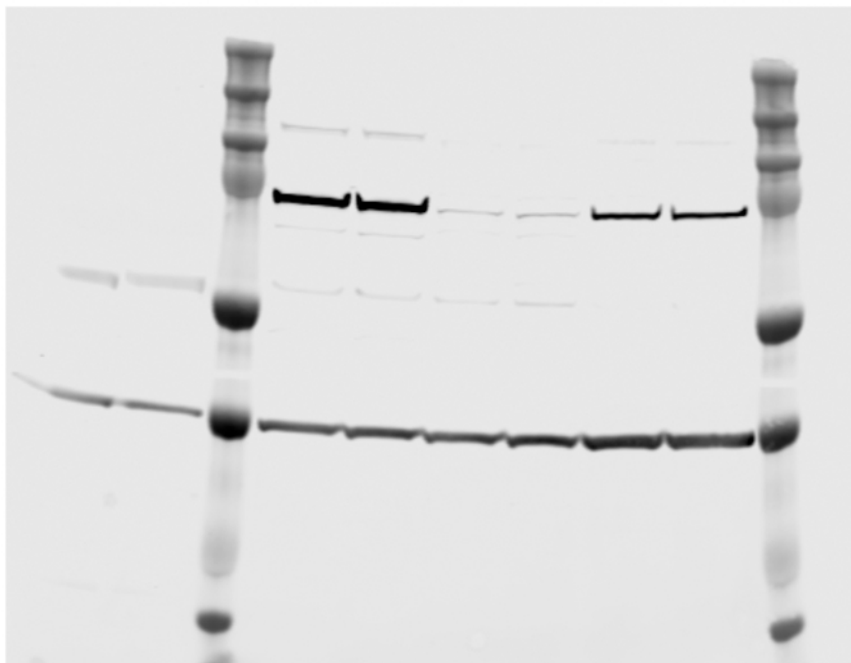

Supplement: S1 Raw images — (PDF) [file pone.0230354.s013.pdf]
