# Supplementary material for: SULF1 suppresses Wnt3A-driven growth of bone metastatic prostate cancer in perlecan-modified 3D cancer-stroma-macrophage triculture models
Source: PLoS One. 2020 May 15;15(5):e0230354. doi: 10.1371/journal.pone.0230354 (PMC7228113; doi:10.1371/journal.pone.0230354)
Supplement: Attachment — Submitted filename: Response to Reviewers.pdf [file pone.0230354.s014.pdf]

UTHealth™
The University of Texas
Health Science Center at Houston
School of Dentistry
Department of Diagnostic & Biomedical Sciences

April 22, 2020

Daotai Nie, Ph.D.
Academic Editor
PLOS ONE

Re: Revision to manuscript PONE-D-20-05598

Dear Dr. Nie,

On behalf of all the authors, we thank you for the positive comments and enthusiasm about our studies presented in Brasil da Costa et al., "*SULF1 suppresses Wnt3A-driven growth of bone metastatic prostate cancer in perlecan-modified 3D cancer-stroma-macrophage triculture models*". We respectfully submit a revised version of this manuscript that incorporates the suggested changes and provides the additional information requested by you and by the reviewers. We think with these suggestions that the manuscript is greatly improved and we hope that this revised version will be acceptable for publication. The point-by-point responses to each set of comments are provided below:

<u>Editor's comments:</u>

"Please provide additional information about the MDApcA 118B AND 183 PDX cells used in this work, including history, culture conditions and any quality control testing procedures (authentication, characterisation, and mycoplasma testing). For all other cell lines, please provide additional information about any quality control testing procedures (authentication, characterisation, and mycoplasma testing)."

Response: We apologize for this oversight and have added the additional information required in the Methods and Material section describing the use of cell lines. Our cell lines are routinely authenticated and tested for mycoplasma.

"Bone marrow tissue specimens containing PCa tumors were obtained with consent under IRB approved protocols at The University of Texas Health Science Center School of Dentistry. (a) Please amend your current ethics statement to include the full name of the ethics committee/institutional review board(s) that approved your specific study. (b) Once you have amended this/these statement(s) in the Methods section of the manuscript, please add the same text to the "Ethics Statement" field of the submission form (via "Edit Submission")."

Response: Again, thank you for bringing this oversight to our attention. We have added the additional information requested both to the manuscript and to the submission form.

"In the ethics statement in the manuscript and in the online submission form, please provide additional information about the tissue samples used in your study. Specifically, please ensure that you have discussed whether all data and tissue samples were fully anonymized before you accessed them. If

Dr. Mary "Cindy" Farach-Carson
1941 East Road, 4220
Houston, Texas 77054
Phone: 713 486 4438 Fax: 713-486-4416

patients provided written informed consent to have data from their medical records used in research, please specifically include this information."

Response: We have added the additional information requested to this form. We note that we received no identifying information from the clinicians for either the bone marrow specimens nor the histological slides. No information from the EHR was provided. The slides purchased from the commercial supplier provided limited information about patient history, but no identifying information. We do not have access to the consent form used by the commercial supplier.

"*Please ensure that you refer to Figure 6 in your text as, if accepted, production will need this reference to link the reader to the figure."*

Response: We modified the text to reflect the proper reference of figure 6 such that it can be readily linked.

Reviewer's comments:

"The significance of studying SULF1 in a context of Wnt3A is not clear... Better background needs to be provided by the authors."

Response: We apologize for not clearly stating the relevance of Wnt3a in the context of PCa progression and as a choice for our studies with sulfatase 1. We added additional context in the revised introduction, citing two previous papers from collaborators in our program project (Dr. Leland Chung and Dr. Neil Bhowmick). We believe the addition of this additional text should make it clear why we chose Wnt3A.

"Fig.1 – it is concerning that authors were not able to detect SULF1 protein with any of the available SULF1 antibodies."

Response: We agree!! It was very upsetting (and expensive) for us to discover that none of the commercially available antibodies were able to pass our internal quality control studies to which we routinely subject antibodies from external suppliers. As a point of clarification, we did not demonstrate that the antibodies do not detect SULF1 protein, but rather we became convinced that they did not SPECIFICALLY detect SULF1. To illustrate this point, we now provide in the new supplemental material images showing the testing, via Western blot, of all commercially available antibodies against SULF1 generated with unique immunogen sequences. Control samples we used included wild-type fibroblasts and SULF1-knockout fibroblasts. None of the antibodies we purchased detected SULF1 specifically (i.e. no signal in the knockout fibroblasts) and with the expected molecular weight. For this reason, we elected to use the transcript detection methods we report here, which we found to be more accurate and provided the added benefit of telling us which cells were making these products.

"RNA signal is very difficult to visualize; mock controls need to be shown for RISH"

Response: We apologize that it was difficult to visualize the mRNA signal in the figures we provided. Because of the detection method, the signal manifests as small brown dots at sites where cells producing these transcripts are located. This difficulty seeing these dots is a major reason we used the pseudocolor conversion to make it easier to visualize in a publication context. We now provide extra figures and controls in the supplemental data to make it easier and more convincing to see the specific signal we describe in our Results – see new fig. S3 for more information and the requested controls.

Dr. Mary "Cindy" Farach-Carson
1941 East Road, 4220
Houston, Texas 77054
Phone: 713 486 4438 Fax: 713-486-4416

“The presence of macrophages in bone marrow tissue is not surprising and has been shown before; the statement that macrophages accumulate around tumors is too strong based on data provided; lack of CD163 expression in the biomimetic model is concerning. CD163 is an M2 marker known to be expressed by the macrophages in the metastatic microenvironment.”

Response: We apologize if our statement about macrophage infiltration was too strong, and we have adjusted our language to describe our results more accurately. Our intention here was to provide complementary evidence about the phenotype and infiltration of macrophages in human bone samples containing PCa tumors. We acknowledge and reference the prior work of others who have demonstrated macrophage presence in PCa tumors of humans and mouse models. We understand that the lack of CD163 in the hydrogels on the surface may seem concerning. Please note, however, that in the patient samples we detected macrophages that expressed CD163, CD206, or both markers. This data is provided in the supplemental figures. We also note that in the case of animal models or human specimens, the contact times between macrophages and cancer cells are much longer than in our hydrogels, where the cells only see one another for days to weeks. This could contribute to the plasticity we observed in the macrophage phenotypes. We added a sentence describing this limitation to the Discussion of this revised manuscript.

“If macrophage-derived TNFa is suspected to be a key-contributor to SULF1 expression, this needs to be shown. Do TNFa levels in macrophages change in response to interaction with tumor cells or in triculture?”

Response: We thank the reviewer for noting this limitation of our study. Our comment on TNFa was based on our previous work with perlecan expression and a detailed study of the perlecan promoter where TNFa was found to be a critical cytokine in transcriptional regulation (Warren et al, 2014, PMID:24700612]. We also noted that several previous studies have determined that tumor-associated macrophages produce TNFa (Chen et a, 2019, PMID: 30844387; Nakagawa et al, 2007, PMID: 17332918; and Dougherty et al, 1997, PMID: 9022125), making this an obvious hypothesis for us. We have not directly shown this to be the case for SULF1, although we speculated there could be a common mechanism. We have softened the language to reflect these concepts accurately. A systematic study of the regulation of SULF1 expression by macrophage-produced TNFa would be an excellent follow up to this work. However, we believe that determining if TNFa levels change in our tricultures is outside the scope of this present study, especially at this time when the ongoing pandemic has restricted laboratory access.

“Fig 5- why was not staining for macrophage markers performed rather than using sphericity filter?”

Response: This work was performed, and we apologize for not including it. We now include this marker data in the revised supplement.

“Overall contribution of macrophages to tumor cell growth in this model is overstated. The data, as provided, do not convincingly support the conclusions.”

Response: We apologize if we overstated the role of macrophages in our study, and we adjusted our language to reflect our findings more accurately. Changes to the text in the Discussion are noted to soften these conclusions.

“The model is somewhat confusing. An increase of SULF1 expression in the stroma and loss in the tumor cells is an interesting concept that needs to be investigated. Since the analyses of human databases indicate that SULF1 is lost in metastatic tumors (which one would presume contain both tumor cells and the stroma) – how do the data presented here fil with these findings. This disconnect

Dr. Mary “Cindy” Farach-Carson
1941 East Road, 4220
Houston, Texas 77054
Phone: 713 486 4438 Fax: 713-486-4416

between SULF1 levels in the stroma vs. the tumor needs to be better demonstrated by the authors. The experimental evidence provided here is not very strong."

Response: We fully understand this comment, and we think this is one of the most intriguing components of our findings. On initial analysis, we too found the results counterintuitive, but believe we now have a better understanding of these complex interactions that we attempt to convey in the model in Figure 6. We edited the text in the Discussion to better clarify what we believe explains the findings with Wnt3a. SULF1 produced and secreted in the stroma can remove key sites of sulfation needed to sequester Wnt3a, and also it can remove 6-O-sulfate from heparan sulfate co-receptors at the cell surface, removing their co-receptor functions and limiting signaling. Because loss of *SULF1* increases Wnt3a-induced growth, it suggests that the cell surface retention of 6-O-sulfate is critical for growth stimulation. It then makes sense that loss of *SULF1* with progression would increase 6-O-sulfate retention at the cancer cell surface and allow greater receptor signaling to occur.

The human tissues, from which the meta-analysis of the databases (PMID: 27302169) indicated loss of *SULF1* in metastatic tumors, probably do contain both tumor and infiltrated stromal cells, however, the number of tumor cells typically far exceeds the number of stromal cells. This overall reduction of *SULF1* levels is consistent with our findings that SULF1 activity, in total, would be lower as cancer growth accelerates bony at metastatic sites owing to co-receptor activation. A significant advance of our work is the demonstration that the source of this secreted SULF1 is the stroma, not the cancer cells themselves.

We also note that all minor points pointed out by the reviewer comments have been corrected, and we appreciate the feedback that has greatly improved our manuscript.

Sincerely,

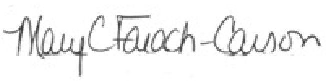

Mary C. (Cindy) Farach-Carson, PhD
Professor and Director, Clinical and Translational Research

Dr. Mary "Cindy" Farach-Carson
1941 East Road, 4220
Houston, Texas 77054
Phone: 713 486 4438 Fax: 713-486-4416
